# Impaired left amygdala resting state functional connectivity in subthreshold depression individuals

**DOI:** 10.1038/s41598-020-74166-x

**Published:** 2020-10-14

**Authors:** Xiaoling Peng, Way K. W. Lau, Chanyu Wang, Lingfang Ning, Ruibin Zhang

**Affiliations:** 1 Cognitive and Neuropsychology Laboratory, Department of Psychology, School of Public Health, Southern Medical University, Guangzhou, 510515 China; 2 Guangzhou Cana School, Guangzhou Rehabilitation and Research Center for Children With ASD, Guangzhou, 510540 China; 3 Department of Special Education and Counselling, The Education University of Hong Kong, Hong Kong, China; 4 Integrated Centre for Wellbeing, The Education University of Hong Kong, Hong Kong, China; 5 Bioanalytical Laboratory for Educational Sciences, The Education University of Hong Kong, Hong Kong, China; 6 Department of Psychiatry, Zhujiang Hospital, Southern Medical University, Guangzhou, 510282 China

**Keywords:** Depression, Amygdala, Limbic system

## Abstract

Subthreshold depression (StD) affects people who experience clinically relevant depressive symptoms, which does not meet the diagnostic criteria for major depressive disorder (MDD). StD represents an ideal model for understanding the pathophysiological mechanisms of depression. Impaired emotion processing is a core feature of depression; careful investigation is required to better understand the neural correlates of emotion processing in depressed populations. In the current study, we explored whether the resting-state functional connectivity of the amygdala, a hub that taps a wide range of brain areas involved in emotion processing, is altered in individuals with StD when compared with healthy controls. Resting-state imaging data was collected from 59 individuals with StD and 59 age- and gender-matched controls. We found that the resting-state functional connectivity of the left amygdala with the cognitive control network and the left insula was significantly lower in people with StD than that in healthy controls. Such association was not observed in the right amygdala. Furthermore, functional connectivity strength between the left amygdala and the left precuneus was positively associated with depressive symptoms in individuals with StD. Our findings are in line with those reported in subjects with MDD, which may assist in further elucidating the pathophysiological mechanisms of depression, and contribute to the development of tailored treatments for individuals with StD who are at high risk of developing MDD.

## Introduction

Major depressive disorder (MDD), a debilitating psychiatric condition, is a leading cause of mortality worldwide^1^. Several studies have suggested that depression is best explained as a spectrum rather than a collection of discrete categories^2^. Minor and subthreshold depression (StD) affect people who experienced clinically relevant depressive symptoms, which do not meet the diagnosis criteria for MDD. Owing to its high incidence^3^ and the marked negative affect on quality of life of patients^4,5^, StD is increasingly becoming a greater health service burden than MDD. A longitudinal study demonstrated that individuals with StD had a fivefold increased risk of experiencing a first lifetime MDD episode compared to healthy controls^6^. StD is therefore regarded as an ideal model for understanding the pathophysiological mechanisms of depression and aids in the development of tailored treatments for patients with depression at different severity levels.

Deficits in emotion processing are core pathological features of MDD^7^. Specifically, patients with MDD tend to elicit an abnormally high level of negative emotions and abnormally low level of positive emotions^8^. Patients with MDD show attentional biases toward cues for sadness or dysphoria^9^ and have a tendency to interpret neutral or positive information negatively compared with non-depressed individuals^10,11^. Because these negative biases appear to have a key role in the pathophysiology and maintenance of depressive states, further careful investigation is required to better understand emotion processing in depressed populations^12,13^.

Depression-related emotion processing deficits have been correlated with aberrant structure and function in the affective network (AN) of the brain^14^. The amygdala, a critical component of the AN, is a hub that is responsible for a wide range of emotion processing functions, including emotion perception, memory, and regulation^15,16^. Studies have demonstrated that the connectivity of the amygdala-based networks is involved in critical functions relevant to depression including emotion regulation (through connections to the frontal and insular areas), modulation of sensory information (through connections with visual, auditory, gustatory and olfactory cortices), and processing of visceral information related to emotion stimuli (through connections with the brain stem)^17,18^. Importantly, depression would increase amygdala reactivity, which biases towards faster processing of negative emotion stimuli across high-order cortical areas that are involved in more complex processing^19^. Furthermore, depressive symptoms might sensitize affected individuals to both social rejection and social acceptance in everyday experiences^20^, which is associated with an increased neural reactivity of the amygdala^21^. Interestingly, there is evidence to support that neurofeedback from neural activity of the amygdala not only substantially reduces depressive symptoms, but also predicts reduction of depressive symptoms^22,23^.

The aim of the current study was to explore whether resting-state functional connectivity (rs-FC) of the amygdala was altered in individuals with StD compared with individuals without depression symptoms. Because the left and right amygdala might be involved in different emotion regulation processes^24^ and exhibit different functional connectivities in MDD^25^, we selected each side of the amygdala as an independent seed region of interest (ROI), and employed whole-brain, voxel-wise functional connectivity analyses to investigate the amygdala functional connectivity pattern in individuals with StD and healthy controls. Considering the close relationship between StD and MDD, we hypothesized that functional connectivity of the amygdala with the frontal and insular areas was impaired in individuals with StD, especially in the emotion regulation system. In addition, we also examined the association between the functional connectivity of the amygdala and self-reported depression symptoms indexed by Beck Depression Inventory within the StD group.

## Results

### Demographic characteristics

There were no significant differences in demographic characteristics, including age, gender, and head motion profile indexed by frame displacement between StD group and the controls (*P*s > 0.05). The StD group showed higher self-reported depressive symptom scores and state-and-trait anxiety scores than the controls (*P*s < 0.001). The detailed data is listed in Table 1.

**Table 1:** Demographics characteristic between controls and subthreshold depression groups (StD).

	Controls	StD	t/X^2^ (p)
Age	19.95 (1.42)	20.12 (1.39)	− 0.65 (0.51)
Gender	28 M/31F	29 M/30F	0.03 (0.85)
BDI	3.48 (1.92)	17.52 (3.43)	− 27.41(p < 0.001)
State Anxiety	36.07 (6.20)	48.79 (6.93)	− 7.60 (p < 0.001)
Trait Anxiety	32.18 (7.26)	43.40 (6.71)	− 10.51 (p < 0.001)
Frame displacement (FD)	0.12 (0.03)	0.12 (0.03)	− 0.68 (0.50)

StD, subthreshold depression; BDI, Beck Depression Inventory.

### Functional connectivity results

**Figure 1:**
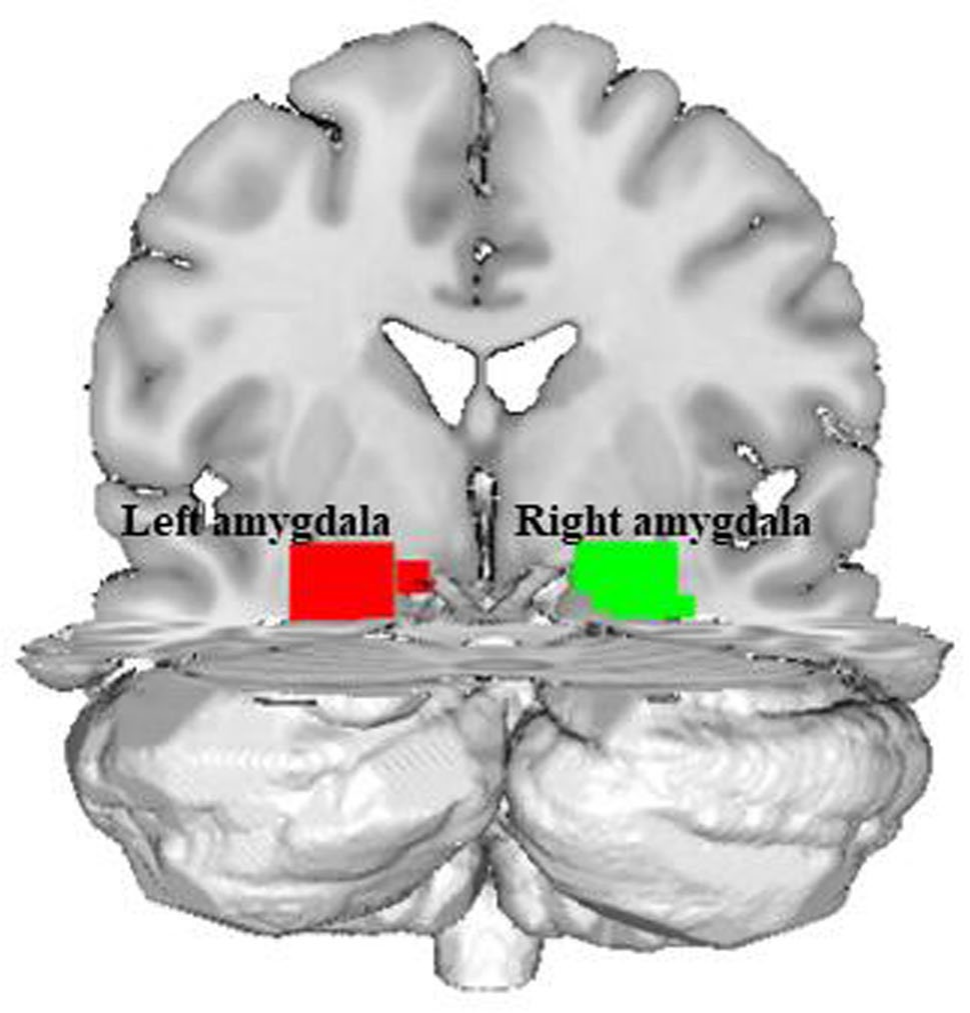
The left and right amygdala regions of interest (red and green, respectively) as defined in the automated anatomical labeling atlas.

**Figure 2:**
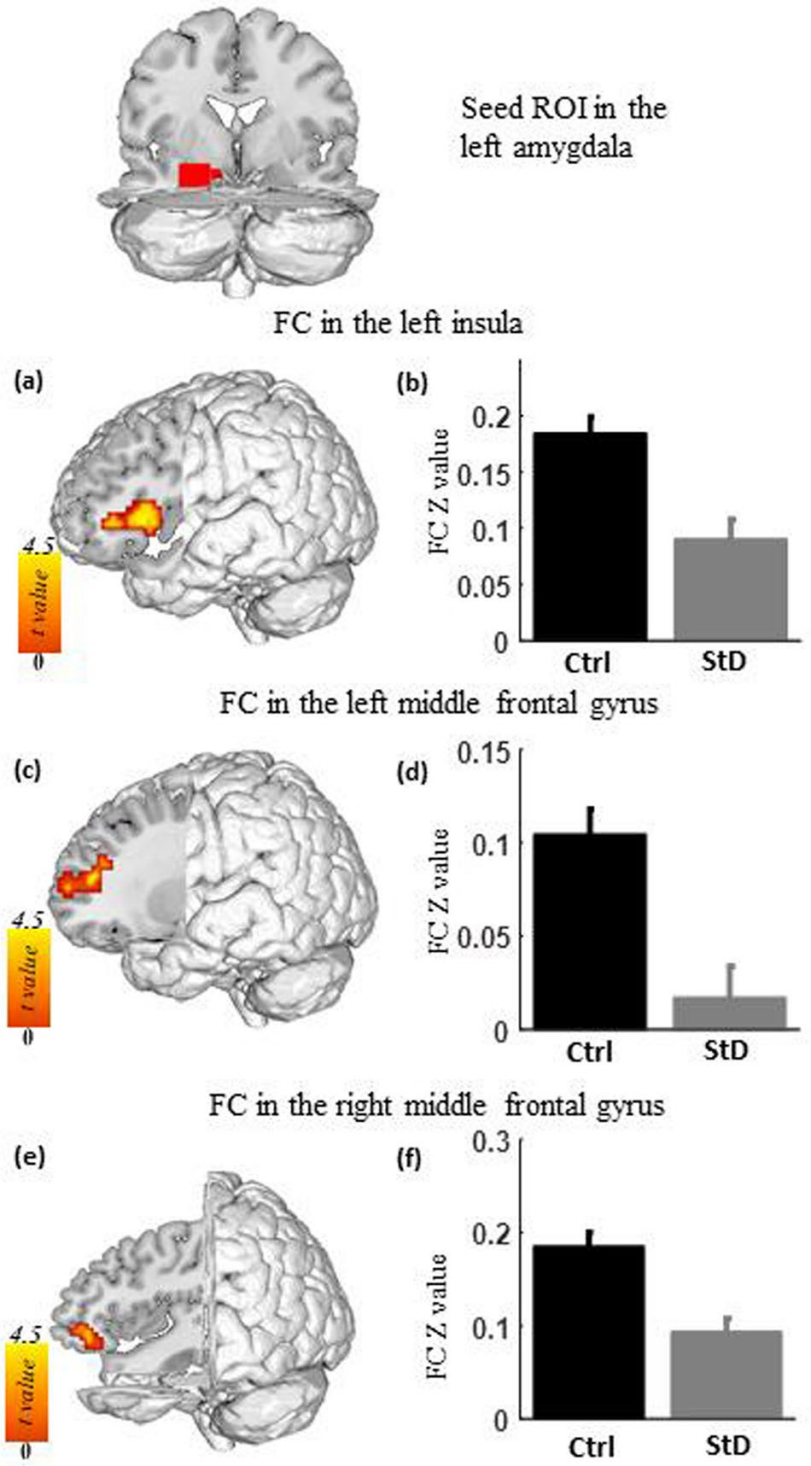
Functional connectivity of the left amygdala in individuals with StD compared with healthy controls (Ctrl). (**a**), (**c**), and (**e**) Compared with the control, the StD group showed decreased functional connectivity between the left amygdala seed region and the left insula (**a**), the left middle frontal gyrus (**c**), and the right middle frontal gyrus (**e**). (**b**), (**d**), and (**f**) Average functional connectivity of the left insula (**b**), the left middle frontal gyrus (**d**), and the right middle frontal gyrus (**f**) in both groups.

Compared with the controls, the StD group exhibited decreased functional connectivity between the left amygdala and regions of the cognitive control network, including the bilateral middle frontal gyri and the salience network, i.e. the left insula (Figs. 1, 2 and Table 2). However, there were no significant differences in the functional connectivity of the right amygdala between the StD group and controls.

**Table 2:** Aberrant functional connectivity of amygdala.

StD compared with controls	Peak coordinate	Peak t	Cluster size (mm^3^)	Cluster information			
				StD		Controls	
				Mean	SD	Mean	SD
***Left amygdala***							
Right middle frontal gyrus	39, 39, − 3	4.31	1215	0.09	0.11	0.19	0.10
Left insula	− 42, 18, 3	4.17	2187	0.09	0.13	0.18	0.11
Left middle frontal gyrus	− 27, 48, 12	4.15	1431	0.02	0.12	0.10	0.11
				StD			
Correlation with BDI				*r*			
Left precuneus	− 3, − 63, 66	4.38	3537	0.52			
***Right amygdala***							
None							

StD, subthreshold depression; SD, standard deviation; BDI, Beck Depression Inventory.

**Figure 3:**
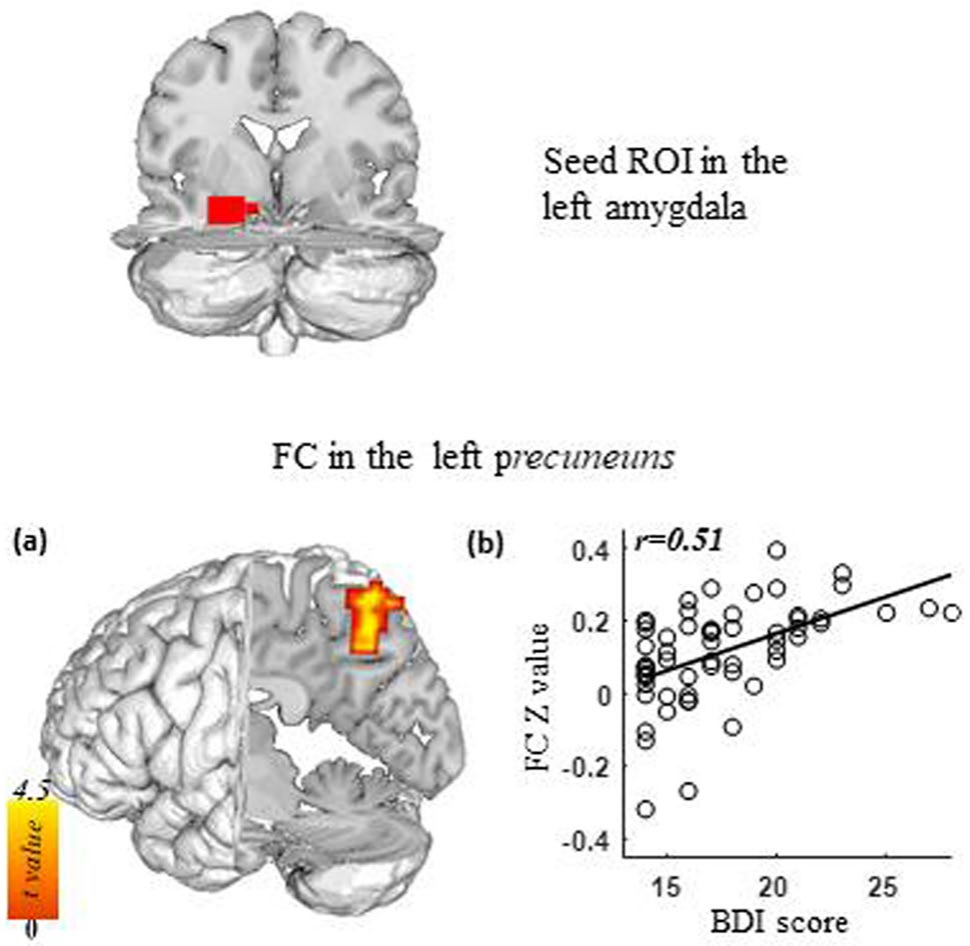
Functional connectivity of the left amygdala as a function of depression severity as indexed by BDI scores. (**a**) For individuals with StD, higher BDI scores were associated with increased functional connectivity between the left amygdala and left precuneus. (**b**) Correlation between BDI and strength of functional connectivity between the left amygdala and left precuneus.

We examined the association between the depressive symptoms indexed by BDI and the functional connectivity of the amygdala within the StD group. We found that depressive symptom severity was positively associated with functional connectivity between the left amygdala and left precuneus (maximum *r* = 0.51, Fig. 3). However, there were no significant associations between the functional connectivity map of the right amygdala and BDI scores.

### Supplementary analysis

We repeated the analysis after regressing out the global signal during data preprocessing, and found that clusters in the left insula still showed significant decreased functional connectivity with the left amygdala in the StD group compared with the controls (Supplementary Fig. S1) and the depressive symptom severity was associated with stronger connectivity between the left amygdala and left precuneus (Supplementary Fig.S2).

## Discussion

In this study, we characterized the functional connectivity profile of the amygdala, a key region for emotion processing, in 59 individuals with StD and 59 age- and gender-matched controls. We found that the rs-FC of the left amygdala with the bilateral middle frontal gyri and the left insula showed a significant decrease in individuals with StD compared with that in healthy controls, which was not observed in the right amygdala. Moreover, the rs-FC between the left amygdala and left precuneus was positively associated with the BDI scores in individuals with StD. Our study demonstrated that, compared with healthy controls, the rs-FC between the amygdala and prefrontal cortex that is responsible for cognitive control, was disrupted in subjects with StD, while hyperconnectivity between the left amygdala and left precuneus was correlated with depressive symptom severity.

The bilateral middle frontal gyri are the core areas of the cognitive control network^26,27^, which is involved in cognitive and executive functions. Decreased amygdala functional connectivity within the cognitive control network, as well as imbalanced amygdala rsFC (both hyper and hypo-connected) within the default mode network have been observed in patients with MDD^25^. According to the cognitive model of depression^28–30^, patients with MDD incline to become trapped in cognitive dysregulation (e.g., memory impairment, difficulty making decisions, and loss of cognitive flexibility), negative attention focus, and negative rumination. Several studies have demonstrated that the middle frontal gyrus, part of the cognitive control network, is involved in top-down cognitive control^31,32^. Decreased middle frontal gyrus activation has been observed in patients with MDD when performing cognitive inhibitory processing during emotion processing^33^, suggesting that these patients have cognitive vulnerability to depression^34^. Importantly, Pannekoek et al.^35^ found that impaired functional connectivity between the amygdala and the cognitive control network could dysregulate top-down cognitive control from the prefrontal cortex to the amygdala and explain affective cognition processing deficits among MDD patients. Notably, at behavioral level, individuals with StD showed emotion processing deficits characterized by spending more time on negative faces and producing less accurate responses compared to controls^36^. At the neural level, individuals with StD showed a significant decreased functional connectivity in the cognitive control network, especially the functional connectivity of the dorsolateral prefrontal cortex with the insula and the regions associated with the temporo-parietal junction compared with controls^37^. Additionally, Li et al.^38^ found that the failure of successful response inhibition could lead to a reduced activation of the prefrontal cortex in response to negative stimuli in individuals with StD. Therefore, impaired connectivity between the left amygdala and bilateral middle frontal gyri could help explain why individuals with StD show cognitive inhibition deficits in emotion processing.

In addition to the aberrant connectivity between the amygdala and the cognitive control network, we also found that compared with controls, individuals with StD exhibited decreased functional connectivity between the left amygdala and the left insula. The insula is a region that underpins the processing of interoceptive states^39^, and has functional interconnections to regions associated with the experience of emotion^40^, hubs of the default mode, cognitive control, and frontostriatal networks^41^. Hence, it is crucial to generate a current emotion awareness state for the integration of stimulus-driven, bottom-up interoceptive signals with top-down predictions. Notably, patients with MDD exhibit heightened interoceptive awareness, which affects their ability to filter both exogenous and endogenous stimuli for adaptive regulation, such as an increase in negative self-focused thought, or rumination that impairs the shifting from internally focused to externally focused attention in individuals with MDD. Studies have suggested that aberrant functional connectivity between the amygdala and insula can lead to stronger anticipation of negative events and a tighter functional link between visceral perception and emotional response^42^. He et al.^43^ also demonstrated that individuals with StD presented negative anticipation and negative attention bias. Moreover, longitudinal studies have also demonstrated that individuals at ultra-high risk of developing an affective disorder, and who subsequently transition to an affective disorder, have reduced volumes in the insula and amygdala, thereby providing further evidence that these regions may be trait markers for affective disorders^44^. Our finding suggests the decreased amygdala–insula functional connectivity at rest might play a role for maladaptive coupling of emotion processing and autonomic regulation in StD.

We also found that the strength of the functional connectivity between the amygdala and left precuneus positively correlated with the BDI scores. The precuneus is linked to reflective self-awareness^45,46^, and is reliably activated in MDD when instructed with self-related information^14^. Importantly, increased functional connectivity between the amygdala and the precuneus has been frequently reported in MDD (see meta-analysis^25^), and this pattern has been associated with recursive self-referential thinking pattern characterized by increased responses to negative stimuli^47,48^. Our finding that there was a positive association between the amygdala and precuneus indicates that StD may be a suitable model for exploring the pathophysiological mechanisms of depression; moreover, this model may aid in the development of tailored treatments for patients at different stages of the disorder.

Interestingly, we found that the functional lateralization of amygdala connectivity in individuals with StD was limited to the left amygdala. A previous report had indicated that the left amygdala was more frequently activated than the right amygdala during emotion processing^49^. Moreover, Wager et al.^50^ demonstrated that under negative-value emotional stimuli, the neural activity pattern of the amygdala showed lateralization. Although the right amygdala was reported to be activated when encoding an emotional stimulus^51^, different study showed that top-down regulation may involve only the left amygdala, whereas bottom-up responses involve both left and right^52^. Considering the cognitive model of depression^28–30^, our findings further suggest that depressed individuals have cognitive inhibition deficits in emotion processing. Nevertheless, the results of our study suggest that using only the left or the right amygdala as the seed, or combining the results from both sides, may lead to potential differences being overlooked.

In the current study, we only recruited individuals with StD and age- and gender-matched controls to investigate the rs-FC of the amygdala. Although our findings are similar to those observed in MDD, future studies should directly explore potential differences in the amygdala between individuals with StD and MDD. Moreover, studies have suggested that StD may be a risk factor for the development of MDD, it is important to investigate the neural signatures associated with transitioning between mild/moderate depression and severe depression, through a longitudinal study following subjects with StD to recovery or full-blown MDD.

In conclusion, we mapped the functional connectivity profile of the amygdala in individuals with StD, and found that compared with healthy controls, the functional connectivity between the left amygdala and both the cognitive control network and left insula was significantly decreased in individuals with StD, which was not observed in the right amygdala. Moreover, the rs-FC between the left amygdala and the left precuneus was positively associated with the BDI scores in individuals with StD. These findings are similar to those reported in patients with MDD, indicating both emotion processing and emotional regulation deficits in depression.

## Methods

### Participants

All data used in this study were obtained from the Southwest University Longitudinal Imaging Multimodal (SLIM) Brain Data Repository (https://fcon-1000.projects.nitrc.org/indi/retro/southwestuni-qiu-index.html); this is a project focuses on the neuroscience of creativity and affective disorders, and was approved by the Research Ethics Committee of the Brain Imaging Center of Southwest University^53^. Informed written consent was obtained from each participant. The data acquisition protocol was carried out in accordance with the Declaration of Helsinki revised in 1989. All the participants in this project were Chinese undergraduate students with no history of psychiatric or neurological disorders. None of the participants fulfilled the DSM-IV criteria for psychiatric disorders, as assessed by two well-trained and experienced graduate students from the School of Psychology. The participants reported no history of head trauma. On the scanning day, no serious physical illness or use of medication (including antidepressant drugs) that would interfere with brain function was detected. Notably, SLIM data has been used in several scientific studies^54–56^.

In this study, the Beck Depression Inventory (BDI) was used to screen depressive symptoms in this cohort. Among all the participants, StD group comprised 59 young adults (29 males/30 females, mean age 20.12 ± 1.39 years), who exhibited either mild (score of 14–18) or moderate (score of 19–29) depressive symptoms, with a mean score of 17.52 ± 3.43. The control group comprised of 59 age-matched and gender-matched subjects with a mean BDI score of 3.48 ± 1.92 (28 male/31 females, mean age: 19.95 ± 1.42), who were selected from the same database. In addition, state and trait anxiety were also assessed based on the State and Trait Anxiety Inventory^57^. The demographic and clinical characteristics of the participants are summarized in Table1.

### Data acquisition

Imaging data was acquired on a Siemens 3-T MAGNETOM TrioTim System (Siemens, Erlangen, Germany) at West China Hospital of Sichuan University, Chengdu, China. The resting-state BOLD signals were acquired as an 8-min scan of 242 contiguous frames. Data acquisition parameters were as follows: slices = 32; repetition time/echo time = 2000/30 ms; flip angle = 90°; field of view (FOV) = 220 × 220 mm; thickness/slice gap = 3/1 mm; and voxel size = 3.4 × 3.4 × 4 mm^3^. T1 weighted anatomical images were collected, with a recorded repetition time of 1900 ms, an echo time of 2.52 ms, an inversion time of 900 ms, a flip angle of 90°, FOV = 256 × 256, 176 slices with a thickness of 1.0 mm, and a voxel size = 1 × 1 × 1 mm^3^.

### Image preprocessing

Resting state fMRI data was preprocessed using Data Processing & Analysis for (Resting‐State) Brain Imaging (DPABI, https://rfmri.org/dpabi) software^58^ with following steps: discarding the first 10 volumes; slice timing correction; head motion correction; spatially normalized to the Montreal Neurological Institute (MNI) template with the voxel size into 3 mm × 3 mm × 3 mm; spatially smoothing with a 6 mm full width half maximum (FWHM) Gaussian kernel to improve the signal to noise ratio; regressing out the linear trend signal, 24 head motion parameters and the first 5 principal components from a combined white matter/cerebrospinal fluid signals mask with CompCor method^59^; and band-pass filtering (0.01–0.1 Hz) to remove spurious fluctuations in functional connectivity. In addition, scrubbing was performed when Power frame displacement (FD) was found to be greater than 0.5 at a specific time-point. The time points before and after each time-point with FD > 0.5 were scrubbed using each of this time-point with FD > 0.5 as a regressor^60^. Because global signal regression could increase the number of negative functional connectivity and the global signal might be beneficial for understanding clinical populations^61,62^, the global signal was not used as a nuisance regressor in the preprocessing steps.

### Seed based functional connectivity

Functional connectivity analysis for each subject was carried out in DPARSF by applying a seed-based approach. Seeds were derived from the automated anatomical labeling atlas from Wake Forest University PickAtlas toolbox (https://www.nitrc.org/projects/wfu_pickatlas). To address the hemisphere effect, analysis of functional connectivity for the left and right amygdala was performed separately, as previously suggested^63^. Figure 1 shows coronal view of the left and right amygdala ROIs as applied to each of the subject.

The averaged time course was obtained from the seed, and the correlation analysis was performed in a voxel-wise manner to generate the functional connectivity map. The correlation coefficient maps were transformed into Fisher’s Z maps using Fisher's R-to-Z transformation to improve the normality. To investigate functional connectivity of the amygdala at group level, individual Fisher’s Z functional connectivity maps obtained from the functional connectivity analysis in DPARSF were used in the second-level analysis; this was performed by between-group voxel-wise *t*-tests using DPABI. A correlation analysis between BDI scores and functional connectivity of the amygdala at each voxel was conducted to examine the relationship between depression severity and functional connectivity within the StD group. A threshold of voxel-wise *p* < 0.001 and cluster-level *p* < 0.05 family-wise error correction were used for all rs-FC analyses, which is recommended by previous studies^64,65^.

### Ethical approval

This study was approved by the Research Ethics Committee of the Brain Imaging Center of Southwest University.

### Informed consent

All procedures followed were in accordance with the ethical standards of the responsible committee on human experimentation (institutional and national) and with the Helsinki Declaration of 1975, as revised in 2000. Informed consent was obtained from all participants for being included in the study.

## Supplementary information

Supplementary file1
