# Supplementary material for: Impaired left amygdala resting state functional connectivity in subthreshold depression individuals
Source: Sci Rep. 2020 Oct 14;10:17207. doi: 10.1038/s41598-020-74166-x (PMC7560839; doi:10.1038/s41598-020-74166-x)
Supplement: Supplementary file 1 — Supplementary file1 [file 41598_2020_74166_MOESM1_ESM.docx]

*Supplementary*

***Impaired left amygdala resting state functional connectivity in subthreshold depression individuals***

Xiaoling Peng, MSc^1,2+^, Way K. W. Lau, PhD^3,4,5+^, Chanyu Wang, BSc^1^, Lingfang Ning, BSc^1^,^，^ Ruibin Zhang, PhD^1,6*^

^1^Department of Psychology, School of Public Health, Southern Medical University, Guangzhou 510515, China

^2^Guangzhou Cana School (Guangzhou Rehabilitation & Research Center for Children with ASD), 510540, China

^3^Department of Special Education and Counselling, The Education University of Hong Kong, Hong Kong, China

^4^Integrated Centre for Wellbeing, The Education University of Hong Kong, Hong Kong, China

^5^Bioanalytical Laboratory for Educational Sciences, The Education University of Hong Kong, Hong Kong, China

^6^Department of Psychiatry, Zhujiang Hospital, Southern Medical University, Guangzhou 510282, China

^+^ Xiaoling Peng and Way K. W. Lau contributed equally.

* Corresponding author: Ruibin Zhang, Department of Psychology, School of Public Health, Southern Medical University, (e-mail: [ruibinzhang@foxmail.com](mailto:ruibinzhang@foxmail.com))

Running title: Impaired amygdala functional connectivity in subthreshold depression


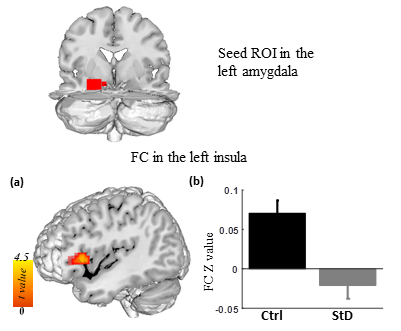


***Figure S1*** Significant reduced functional connectivity strength of the left insula with the left amygdala in StD group compared with controls after regressed out the global signal (a, b).


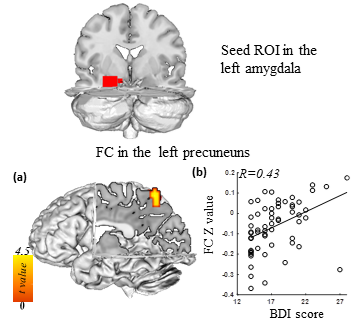


Fig. S2 Functional connectivity of the left amygdala as a function of depression severity as indexed by BDI scores under data preprocessing with global signal regression. (a) For individuals with StD, higher BDI scores were associated with increased functional connectivity between the left amygdala and left precuneus. (b) Correlation between BDI and strength of functional connectivity between the left amygdala and left precuneus.
